# Metabolic phenotype and patient characteristics associate with inter-donor variability of osteogenic differentiation in human adipose stem cells

**DOI:** 10.1016/j.bbrep.2026.102728

**Published:** 2026-07-27

**Authors:** Abdus Sattar, Justus P. Beier, Tim Ruhl

**Affiliations:** Department of Plastic Surgery, Hand Surgery–Burn Center, University Hospital RWTH Aachen, Pauwelsstraße 30, Aachen, 52074, Germany

**Keywords:** Mesenchymal stem cells, Bone tissue engineering, Differentiation, Metabolism, Glycolysis, Mitochondrial membrane potential

## Abstract

Adipose-derived stem/stromal cells (ASCs) are promising candidates for bone tissue engineering due to their abundance, accessibility, and osteogenic differentiation capacity. However, substantial inter-donor variability limits their standardized clinical application. This study investigated whether metabolic characteristics and donor-related parameters are associated with ASC osteogenic differentiation potential. ASCs were isolated from human subcutaneous adipose tissue (n = 26) and cultured under osteogenic conditions. Cell viability was evaluated at days 2 and 14, including cell proliferation (crystal violet staining), overall metabolic activity (resazurin conversion), as well as glucose uptake (2-NBDG) and mitochondrial membrane potential (TMRE). Osteogenesis was quantified by alkaline phosphatase activity, calcium deposition, and secretion of SPARC and osteocalcin (BGLAP). Osteogenic induction significantly enhanced osteogenic markers and mineralization across donors and was accompanied by a distinct metabolic phenotype characterized by reduced per-cell metabolic activity and glucose uptake, and elevated mitochondrial membrane potential. Early (day 2) metabolic parameters did not predict subsequent mineralization capacity, whereas metabolic activity at day 14 correlated inversely with calcium deposition. Donor age, body mass index, and blood glucose levels were not associated with osteogenic outcomes. These findings indicated that inter-donor variability in ASC osteogenesis was accompanied by a characteristic late-stage metabolic signature and suggested that metabolic profiling may support functional characterization of osteogenic differentiation potential.

## Highlights

• ASCs display variable osteogenic differentiation among human donors.
• Early metabolic features (Day 2) do not predict later mineralization capacity of ASCs.
• Day 14 metabolic activity correlates inversely with total calcium deposition of ASCs.
• Donor age, BMI, and glucose do not significantly impact osteogenic outcomes of ASCs.

## 1 Introduction

Large bone defects, delayed unions, and non-unions remain a major clinical challenge. Cell-based bone tissue engineering aims to supplement endogenous repair by providing osteogenic progenitors and pro-regenerative paracrine signals, yet the performance of mesenchymal stem or stromal cells (MSCs) is frequently inconsistent [[1], [2], [3]]. Among MSC sources, adipose stem or stromal cells (ASCs) have attracted considerable interest because they can be harvested in high numbers with comparatively low donor-site morbidity and show osteogenic differentiation potential under standard induction conditions [1,2,4]. Nevertheless, a recurring bottleneck for translational use is the pronounced donor-to-donor variability in ASC growth, metabolism, and differentiation behavior, which limits reproducibility and complicates donor selection for autologous and allogeneic strategies [1,5,6].

Such variability is likely multi-factorial. Donor-associated variables (e.g., age, adipose depot, cellular composition, inflammatory and metabolic status, medication, hormonal levels) can influence ASC biology, while experimental variables (culture conditions, passage number, induction medium, readout methods) further contribute to heterogeneity in cell differentiation. In the specific context of aging, the literature is notably inconsistent: human studies have reported decreased, unchanged, or even increased osteogenic readouts with advancing donor age, often differing by tissue depot, age grouping, osteogenic protocols, and endpoints [7]. This lack of consensus suggests that age alone is an insufficient predictor of ASC performance. Instead, functional potency is likely governed by a complex interplay of donor-specific variables, including systemic inflammation, medication use, and underlying comorbidities such as diabetes or chronic illness, which may be more accurately reflected by the cells' metabolic characteristics [8,9].

Cellular metabolism is increasingly recognized as a regulator and readout of lineage commitment [10]. Under standard conditions (the 'resting' phase), MSCs primarily rely on glycolysis to maintain a low-redox environment and protect against oxidative stress. Following the initiation of osteogenic induction, a distinct metabolic switch occurs during the early commitment phase. This transition is characterized by a shift from glycolytic metabolism to an increased reliance on oxidative phosphorylation (OXPHOS) to meet the heightened energy demands of bone matrix synthesis. Crucially, this switch is often preceded by a period of mitotic expansion, wherein the cells undergo a final round of proliferation (clonogenic expansion) before exiting the cell cycle to commit fully to the osteogenic lineage. This coordinated remodeling of energy demand and substrate use is not merely a byproduct, but a fundamental regulatory driver of mammalian stem cell differentiation and osteoblast maturation [[11], [12], [13], [14], [15], [16], [17]]. Glucose uptake and intracellular glucose handling participate actively in osteogenic signaling by interacting with intracellular signaling pathways (e.g., by influencing AMPK activity and RUNX2-dependent programs) [13,14,18,19]. In parallel, mitochondrial membrane potential reflects mitochondrial activity, *i.e.* indicating OXPHOS, and has been used to characterize differentiation-associated mitochondrial remodeling [11,12]. These observations raise a practical question relevant to ASC-based bone regeneration: can simple, scalable metabolic assays serve as (i) potency-associated signatures during osteogenic differentiation and/or (ii) early predictors of donor-dependent osteogenic outcomes?

In this study, we quantified osteogenic differentiation of ASCs after 14 days using multiple established orthogonal readouts, and tested for correlation against the summed metabolic activity, as well as the detailed glucose uptake and mitochondrial membrane potential at baseline (day 2) and late differentiation (day 14). Following a predefined analysis regime, mineralization was used as the primary osteogenic outcome, and we tested associations with cellular metabolic features and donor characteristics available from clinical records. Our goal was to clarify whether inter-donor variability of ASC osteogenesis was reflected by a measurable metabolic phenotype and whether donor-level variables such as age, BMI, or blood glucose contributed to this variability.

## 2 Materials and methods

### 2.1 Materials

Fetal bovine serum (FBS), high-glucose Dulbecco's modified Eagle's medium (DMEM; 4.5 g/L glucose), DMEM/F-12, low-glucose DMEM, trypsin–EDTA, and 2-(7-nitro-2,1,3-benzoxadiazol-4-yl)-d-glucosamine (2-NBDG) were purchased from Thermo Fisher Scientific (Darmstadt, Germany). Collagenase type I was obtained from Worthington Biochemical Corporation (Lakewood, NJ, USA). Ascorbate-2-phosphate, β-glycerophosphate, Alizarin Red S, paraformaldehyde (PFA), dexamethasone, bovine serum albumin (BSA), Tween®20, KH_2_PO_4_, CaCl_2_, NaHCO_3_, 8-hydroxyquinoline, cresolphthalein complexone, 2-amino-2-methyl-1,3-propanediol (AMPED) and 2-amino-2-methyl-1-propanol (AMP) were obtained from Sigma-Aldrich Chemie GmbH (Taufkirchen, Germany). Resazurin was obtained from Santa Cruz Biotechnology (Heidelberg, Germany). Formaldehyde solution, DMSO, HCl, NaOH, p-nitrophenyl phosphate (pNPP), KCl, and the salts MgCl_2_, Na_2_HPO_4_, and MgSO_4_ were purchased from Carl Roth GmbH + Co. KG (Karlsruhe, Germany). Insulin and protease inhibitor were obtained from Roche Diagnostics GmbH (Mannheim, Germany). HEPES, isopropanol, and Oil Red O were purchased from Merck KGaA (Darmstadt, Germany). Phosphate-buffered saline (PBS) was purchased from Biochrom GmbH (Berlin, Germany). Human osteocalcin (Bone Gamma-carboxyglutamate Protein, BGLAP) and SPARC (Secreted Protein Acidic and Rich in Cysteine) DuoSet ELISA kits were obtained from Bio-Techne GmbH (R&D Systems; Wiesbaden-Nordenstadt, Germany). Tetramethylrhodamine ethyl ester (TMRE) was purchased from MedChemExpress LLC (Monmouth Junction, NJ, USA). Phenylmethylsulfonyl fluoride (PMSF) was purchased from AppliChem GmbH (Darmstadt, Germany). Sodium chloride (NaCl) was purchased from KMF Laborchemie-Handels GmbH (Lohmar, Germany).

### 2.2 Study design

This study was conducted from January 2022 to December 2024. Human subcutaneous adipose tissue was obtained from 26 donors (24 females, 2 males) who underwent abdominoplasty or liposuction procedure (Table 1). The study was approved by the regional ethics committee (EK163/07) and conformed to the principles outlined in the Declaration of Helsinki. All participants provided their written informed consent to participate in this study and the privacy rights of the subjects were observed.

**Table 1:** Donor (patient) characteristics.

Serial no.	Age (years)	Sex	Anatomical origin	BMI (kg/m^2^)	Blood glucose (mg/dL) *
1	53	F	Abdomen	35.06	138
2	42	F	Abdomen	29.05	109
3	52	F	Abdomen	33.87	126
4	35	F	Abdomen	35.20	84
5	62	F	Abdomen	31.64	-
6	57	F	Abdomen	20.94	89
7	26	M	Abdomen	23.37	93
8	47	F	Abdomen	23.71	83
9	44	F	Liposuction	30.48	90
10	59	F	Abdomen	33.90	89
11	56	F	Abdomen	25.28	74
12	48	F	Abdomen	22.94	80
13	46	F	Abdomen	32.24	91
14	35	F	Liposuction	31.86	147
15	53	F	Abdomen	35.06	81
16	27	F	Abdomen	27.78	93
17	46	F	Abdomen	24.46	112
18	38	F	Abdomen	23.71	74
19	45	F	Abdomen	26.18	144
20	61	F	Abdomen	34.06	85
21	29	F	Abdomen	26.81	77
22	58	F	Liposuction	27.55	106
23	n.a.	F	Liposuction	24.46	77
24	34	M	Abdomen	34.60	83
25	48	F	Abdomen	28.09	107
26	66	F	Abdomen	37.89	102
Median (IQR)	47 (38–56)	—	—	28.57 (24.66–33.89)	90 (83–107)
Mean ± SD	46.7 ± 11.3	—	—	29.2 ± 4.8	97.4 ± 21.6

Footnote: Sex is coded as F (female) and M (male). BMI was calculated as kg/m^2^. n.a., not available. Summary statistics (median/IQR and mean/SD) were calculated excluding missing values. *Blood glucose values were extracted from routine clinical records and represent random (non-fasted) measurements.

### 2.3 Isolation and culture of ASC

The fat tissue was minced and incubated for 45 min at 37 °C with continuous shaking in 0.2% collagenase solution at a volume ratio of 1:1. The digested fat solution was filtered through a 250 μm mesh and centrifuged at 300x *g* for 10 min. The upper oil and middle aqueous phases were discarded and the cell pellet was re-suspended in 30 mL PBS followed by centrifugation at 300x g for 10 min. The supernatant was discarded and the remaining cells were seeded in proliferation media containing DMEM/F-12 supplemented with 10% FBS, 0.1% basic fibroblast growth factor (bFGF) and 0.5% penicillin-streptomycin. The cells have been identified as ASCs by their characteristic plastic adherence and their ability to differentiate towards the chondrogenic, osteogenic, and adipogenic lineage, as described earlier [3,20]. Cells were expanded at standard culture conditions and split after reaching 80% confluence. Experiments were performed with cells from passages P1 and P2 [21].

### 2.4 Osteogenic differentiation of ASCs

ASCs (30,000 cells/cm^2^) were seeded in 12-well plates containing proliferation media without antibiotics. After 2 days, when cells reached 80% confluence, the media was replaced with osteogenic media containing DMEM low glucose, 10% FBS, 200 μM l-Ascorbic acid-2-phosphate, 100 nM dexamethasone, and 10 mM β-glycerophosphate. The medium was replaced with fresh medium every second day of differentiation for 14 days [21].

### 2.5 Gross metabolism by resazurin conversion

To comprehensively characterize cellular metabolism, this study employed three complementary techniques: resazurin conversion, 2-NBDG uptake, and TMRE quantification. Resazurin conversion reflects the summed cellular metabolism by measuring the total cellular reducing capacity driven by various cytoplasmic and mitochondrial enzymes. This global metabolic readout differs from 2-NBDG uptake, which specifically targets glucose transport as a surrogate for glycolytic flux, and TMRE quantification, which measures mitochondrial membrane potential as a direct indicator of mitochondrial activity for oxidative phosphorylation (OXPHOS) [[22], [23], [24]].

The summed/total metabolic activity of ASCs was measured by conversion of resazurin at day 2 (baseline) and day 14 (differentiated) as described earlier [25]. After 1 h of incubation in 10% of a resazurin stock solution (0.1 mg/mL PBS) at 37°C, 5% CO_2_ in a humidified atmosphere, 100 μL of the medium were carefully transferred into a 96-well plate and fluorescence was measured in triplets in a microplate reader (BMG Labtech, Ortenberg, Germany) at wavelength of 590 nm (excitation 544 nm).

### 2.6 Cell proliferation by crystal violet stain

Crystal violet (CV) staining was performed at day 2 and 14 following the protocol described earlier [26]. Briefly, ASCs were washed with PBS and fixed in 2-propanol for 10 min at room temperature. Following extensive washing with PBS containing 0.05% Tween20, cells were stained with a crystal violet solution (0.1% crystal violet in aqua bidest) for 20 min. After removal of the crystal violet solution, plates were washed with aqua bidest. Adsorbed crystal violet was washed out in 500 μL acetic acid (33%) per well and incubated for 15 min with gentle agitation. Finally, 70 μL per sample were transferred in triplets to an optical plate and absorbance was quantified at 620 nm.

### 2.7 Glycolysis by 2-NBDG uptake

Cellular glucose uptake was assessed using the fluorescent glucose analog 2-NBDG in a 96-well format. The assay was performed following the protocol described earlier with slight modifications [23,27]. ASCs were seeded in 96-well plates (6,000–9,000 cells/well). A 200 μM 2-NBDG working solution was prepared by diluting 400 μL of a 5 mM stock into 10 mL Hanks' Balanced Salt Solution (HBSS). Cells were washed twice with PBS and pre-incubated with insulin (1 μM in HBSS) for 30 min at 37°C to stimulate glucose transport. The insulin solution was then replaced with 100 μL of 200 μM 2-NBDG per well and plates were incubated for 24 h at 37°C in a humidified incubator. Vehicle was the same buffer without 2-NBDG; blank wells contained PBS only. Following incubation, the cells were washed twice with PBS (5 min per wash) to eliminate any non-internalized probe. Intracellular fluorescence was subsequently measured in 100 μL of fresh PBS using a microplate reader at excitation and emission wavelengths of 540/570 nm, respectively. To ensure accurate comparisons across donors and experimental conditions, all fluorescence signals were normalized to cell number based on the optical density obtained from the crystal violet assay.

### 2.8 Mitochondrial activity by TMRE uptake assay

Mitochondrial membrane potential was assessed using TMRE in a plate-based assay following the protocol described by Crowley et al. with slight modifications [24]. Cells were seeded in microplates and allowed to adhere. Culture medium was removed and cells were washed with PBS. TMRE was prepared at 5 μM in HBSS and added to the wells. Incubation is typically performed for 1–2 h, however, in ASCs this incubation time yielded insufficient signal-to-noise in our plate-reader format. Therefore, to obtain a robust uptake signal for our conditions, plates were incubated with TMRE for 24 h at 37°C in a humidified incubator. After incubation, the staining solution was aspirated and cells were washed twice with PBS containing 0.2% BSA. Fluorescence was measured at Ex/Em 549/575 nm. TMRE signals were normalized to crystal violet optical density to account for differences in cell number.

### 2.9 Alkaline phosphatase (ALP) activity assay

After 14 days of osteogenic induction, cells were washed with PBS and treated with lysis buffer solution (1% Tween 20 in aqua bidest, pH = 9). Cells were then incubated in Phenylmethylsulfonylfluoride (PMSF) for 10 min at room temperature [28]. ALP substrate solution (10 mM pNPP, 100 mM AMPED, and 5 mM MgCl_2_, pH = 9.5) was added and incubated at 37 °C for 1 h. The reaction was stopped by adding 2 M NaOH. Afterwards, 200 μL supernatant was transferred to a 96-well plate in triplets and absorbance was measured at 405 nm [29]. Values were normalized by optical density of crystal violet assay.

### 2.10 Cresolphthalein assay

To determine extracellular matrix calcification as the late osteogenic marker, the cresolphthalein assay was performed after 14 days of osteogenic differentiation [28]. First, ASCs were washed with PBS and then fixed with 4% PFA for 20 min, followed by washing with aqua bidest. Subsequently, the acidic cresolphthalein solution (0.1% ortho‐cresolphthalein‐complexone, 1% 8‐hydroxychinoline in 30 mL of 37% HCl, diluted in 500 mL aqua bidest) was added and incubated for 5 min, followed by alkaline 2-amino-2-methyl-1-propanol (AMP) buffer (76 mL AMP in 500 mL aqua bidest, pH = 10.7 with HCl). Finally, 100 μL colored solution was transferred to a 96-well plate in triplicate and the absorbance of the cresolphthalein complex was measured at 580 nm [30].

### 2.11 Alizarin Red staining for matrix mineralization

For Alizarin Red S staining, cells were washed with PBS and fixed with 4% paraformaldehyde (PFA) for 20 min at room temperature [28]. After fixation, cells were washed with distilled water and stained with 0.5% Alizarin Red S solution (sterile-filtered immediately before use). 300 μL of the filtered staining solution was added per well, followed by incubation for 20 min at 37°C. Cells were then washed five times with distilled water until the washing solution remained clear. Following air-drying of the plates, the mineralized matrix (calcium deposition) was documented using an EVOS® FL Auto Imaging System (Thermo Fisher Scientific, Darmstadt, Germany).

### 2.12 SPARC and Osteocalcin (BGLAP) quantification by ELISA

SPARC and osteocalcin (BGLAP) levels in supernatants of cells differentiated for 14 days were measured by ELISA according to the manufacturer protocol (R&D Systems). Briefly, the ELISA grade 96-well plate was coated with capture antibody and incubated at room temperature for 24 h. The next day, the unspecific binding site was coated with reagent diluent (5% Tween20 in PBS, pH 7.2-7.4). After that, samples and standards were added and incubated for 2 h, followed by incubation with detection antibody for 1 h. Next, Streptavidin-HRP was added for 20 min followed by the addition of substrate solution. After 20 min, the reaction was stopped by the addition of a stop solution (2 N H_2_SO_4_). Each step was followed by washing the plate 3x with washing buffer (0.05% Tween20 in PBS, pH 7.2-7.4). Finally, the absorbance was measured at 450 nm.

### 2.13 Clinical donor characteristics

Anonymous donor characteristics were retrieved from medical records (where available), including age, sex, anatomical origin of tissue, BMI, blood pressure, waist circumference, and metabolic laboratory values (Table 1). Blood glucose levels represent random (non-fasted) values measured 1–2 days prior to the surgical procedure.

### 2.14 Data processing and statistical analysis

All statistical analyses were performed, figures and statistical graphs were generated using GraphPad Prism version 10.6 (GraphPad Software, Boston, MA, USA). Adobe Photoshop CS6 (Adobe Inc., San Jose, CA, USA) was used to process digital images and adjust figure layouts. Functional readouts were normalized to crystal violet optical density to account for differences in cell number. Shapiro-Wilk testing indicated that the data were not-normally distributed, thus, results were statistically analyzed using non-parametric testing. Parallel, time-matched paired conditions within the same donor at day 14 were compared using the Wilcoxon matched-pairs signed-rank test to account for the shared biological background. For multi-group evaluations across day 2 baseline, day 14 control, and day 14 osteogenic conditions, the Friedman test followed by Dunn's multiple-comparison post-hoc correction was used to control for donor-level baseline variance within the matched triplets. Unpaired comparisons between stratified low- and high-osteogenic responder subgroups were analyzed using the Mann-Whitney *U* test. Data were presented as box-and-whisker plots, with the horizontal line representing the median and the box boundaries indicating the 25th and 75th percentiles. Whiskers were calculated using the Tukey method, and individual data points were plotted to show the potential outliers (if any).

Associations between the primary osteogenic outcome (calcium deposition quantified by the o-cresolphthalein assay) and cellular or donor characteristics were evaluated using two-tailed Spearman rank correlation. Statistical significance was defined as p < 0.05.

## 3 Results

### 3.1 Primary human ASCs exhibit pronounced inter-donor variability in late-stage mineralization capacity

**Fig. 1:**
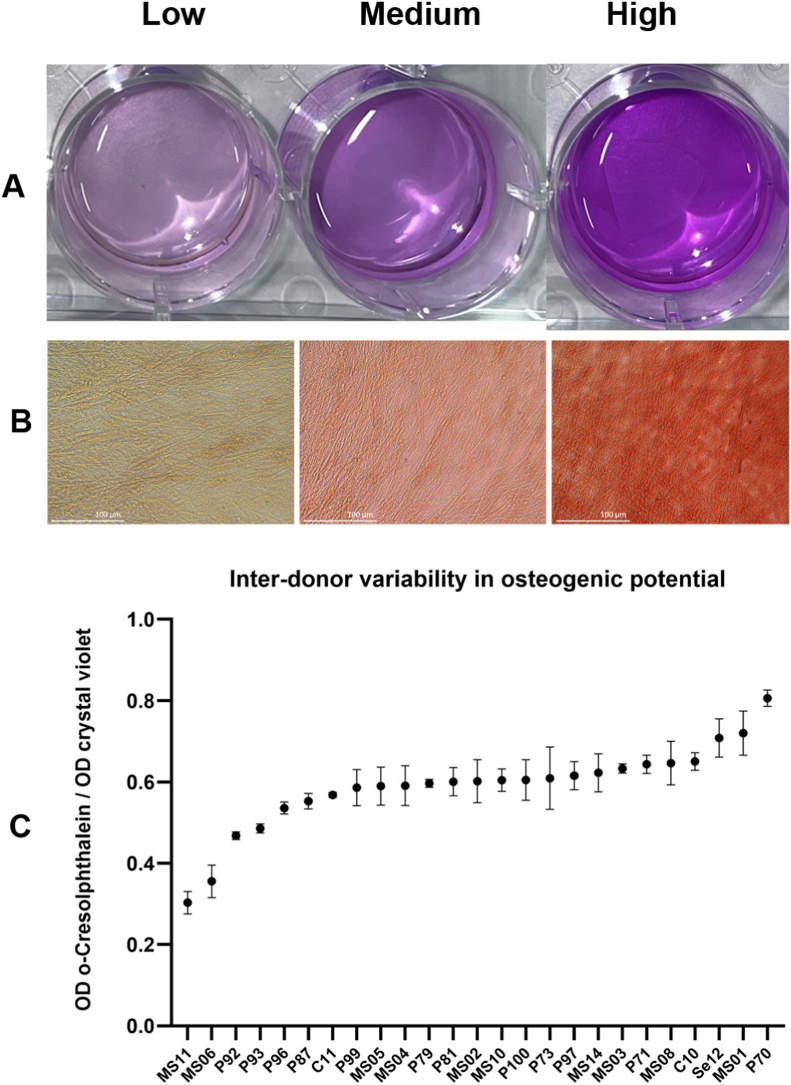
**Visual and quantitative characterization of inter-donor variability in late-stage ASC mineralization.** (A–B) Representative images from three distinct donors demonstrating a visual gradient of low (left), medium (middle), and high (right) mineralization outcomes after 14 days of culture: (A) macroscopic o-cresolphthalein staining targeting calcification and (B) microscopic Alizarin Red S staining for matrix mineralization (scale bars = 100 μm). (C) Quantitative inter-donor variability in osteogenic potential across the cohort (n = 25 donors). Individual donors are rank-ordered by late-stage calcium deposition (normalized to crystal violet optical density) following 14 days of osteogenic induction. Data are presented as mean ± SD.

To evaluate the baseline heterogeneity of functional matrix mineralization among primary human adipose-derived stem/stromal cells (ASCs), visual and quantitative assessments were performed across the donor cohort following 14 days of osteogenic induction. Pronounced donor-to-donor variability was visually observed in terminal osteogenic endpoints, as evidenced by a clear staining gradient for extracellular matrix calcification via the o-cresolphthalein assay (Fig. 1A) and matrix mineralization via Alizarin Red S staining (Fig. 1B). This qualitative heterogeneity was confirmed by quantitative evaluation of matrix calcification, where normalized calcium deposition values varied substantially across individual lines (Fig. 1C).

### 3.2 Osteogenic induction produces robust osteogenic differentiation and a distinct metabolic phenotype

**Fig. 2:**
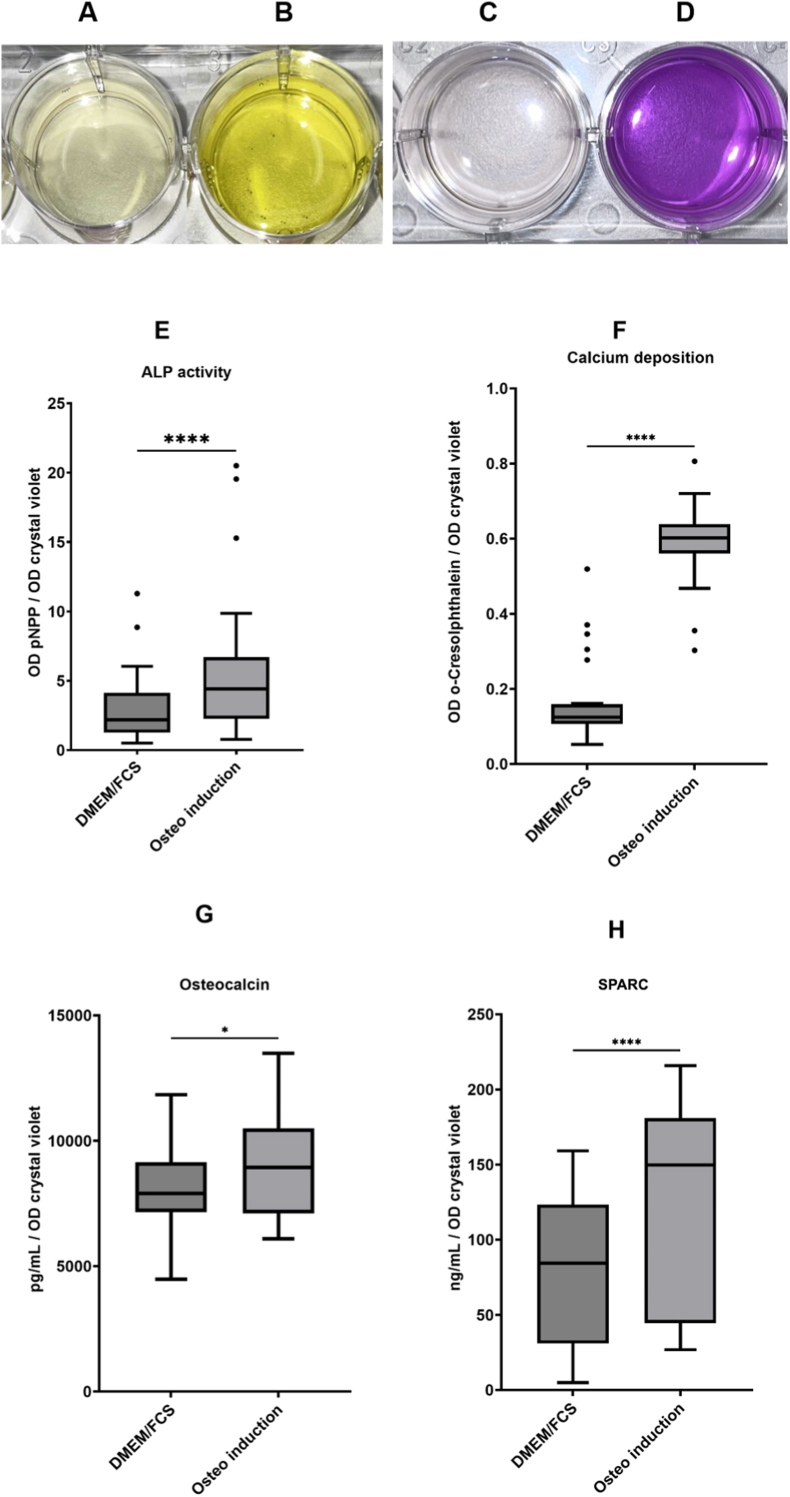
Osteogenic differentiation profiling of ASCs across donors. Representative photos of ALP staining (A, control; B, osteogenic induction) and cresolphthalein assay targeting calcification (C, control; D, osteogenic induction). (E) ALP activity (n = 26), (F) calcium deposition (n = 25), and secreted (G) osteocalcin (BGLAP) and (H) SPARC (ELISA; n = 24) at day 14 control vs day 14 osteogenesis. Statistics: Wilcoxon matched-pairs signed rank test. Data were normalized to cell numbers (optical density of crystal violet). Individual points represent outliers. *p < 0.05, **p < 0.01, ***p < 0.001, ****p < 0.0001.

Across the donor cohort, osteogenic induction elicited a robust and significant differentiation response compared to cells maintained in control medium (DMEM/F-12 supplemented with 10% FBS). This osteogenic commitment was qualitatively evidenced by a marked increase in alkaline phosphatase (ALP) activity (Fig. 2A and B) and extracellular matrix calcification (Fig. 2C and D). Quantitative assessment further confirmed this transition, showing significantly elevated enzymatic ALP activity (Fig. 2E) and a substantial increase in calcium deposition as measured by the o-cresolphthalein assay (Fig. 2F). Furthermore, the maturation of the osteogenic phenotype was supported by the significantly increased secretion of the bone-specific proteins Osteocalcin (Fig. 2G) and SPARC (Fig. 2H) into the culture supernatant.

**Fig. 3:**
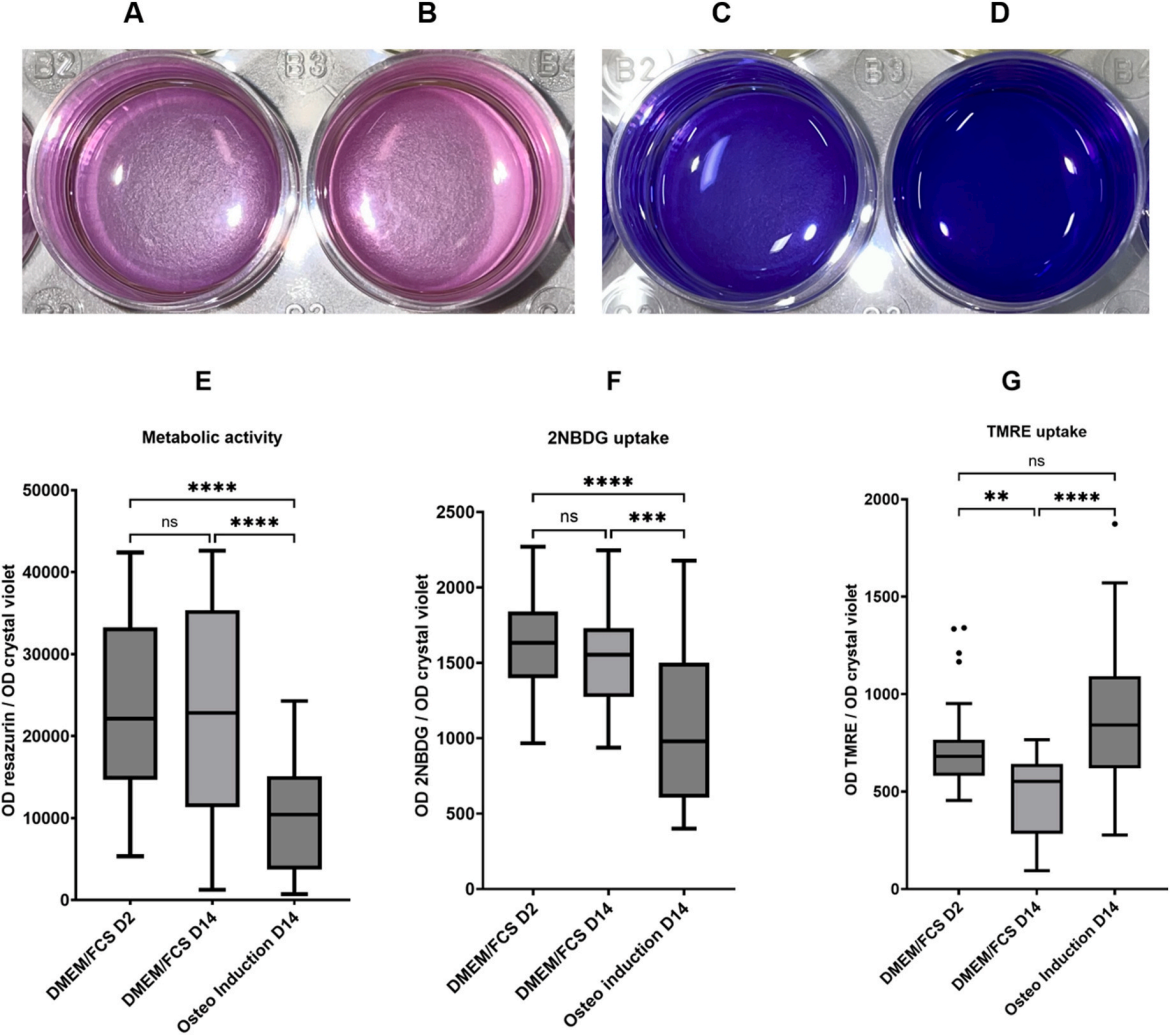
Representative photos of (A-B) Resazurin staining (A, control; B osteogenic induction) and (C-D) crystal violet staining (C, control; D, osteogenic induction). (E-G) Metabolic profiling of ASCs across donors. (E) Relative metabolic activity, (F) glucose uptake surrogate and (G) mitochondrial membrane potential (TMRE) at day 2 control, day 14 control, and day 14 osteogenic induction (n = 26). Statistics: Friedman test with Dunn's multiple comparisons. Data were normalized to cell numbers (optical density of crystal violet). Individual points represent outliers. *p < 0.05, **p < 0.01, ***p < 0.001, ****p < 0.0001.

Metabolic profiling normalized to cell number revealed a distinct phenotype under osteogenic conditions. Crystal violet absorbance indicated increased cell numbers at day 14 under osteogenic induction compared with controls, whereas per-cell metabolic activity (resazurin/crystal violet) and per-cell glucose uptake (2-NBDG/crystal violet) were reduced at day 14 compared with day 2 and day 14 control conditions (Fig. 3E and F). TMRE signal decreased from day 2 to day 14 under control conditions, but remained higher under osteogenic induction compared with day 14 controls, consistent with osteogenesis-associated changes in mitochondrial membrane potential (Fig. 3G**).**

### 3.3 Baseline (day 2) metabolic features do not predict later mineralization

**Fig. 4:**
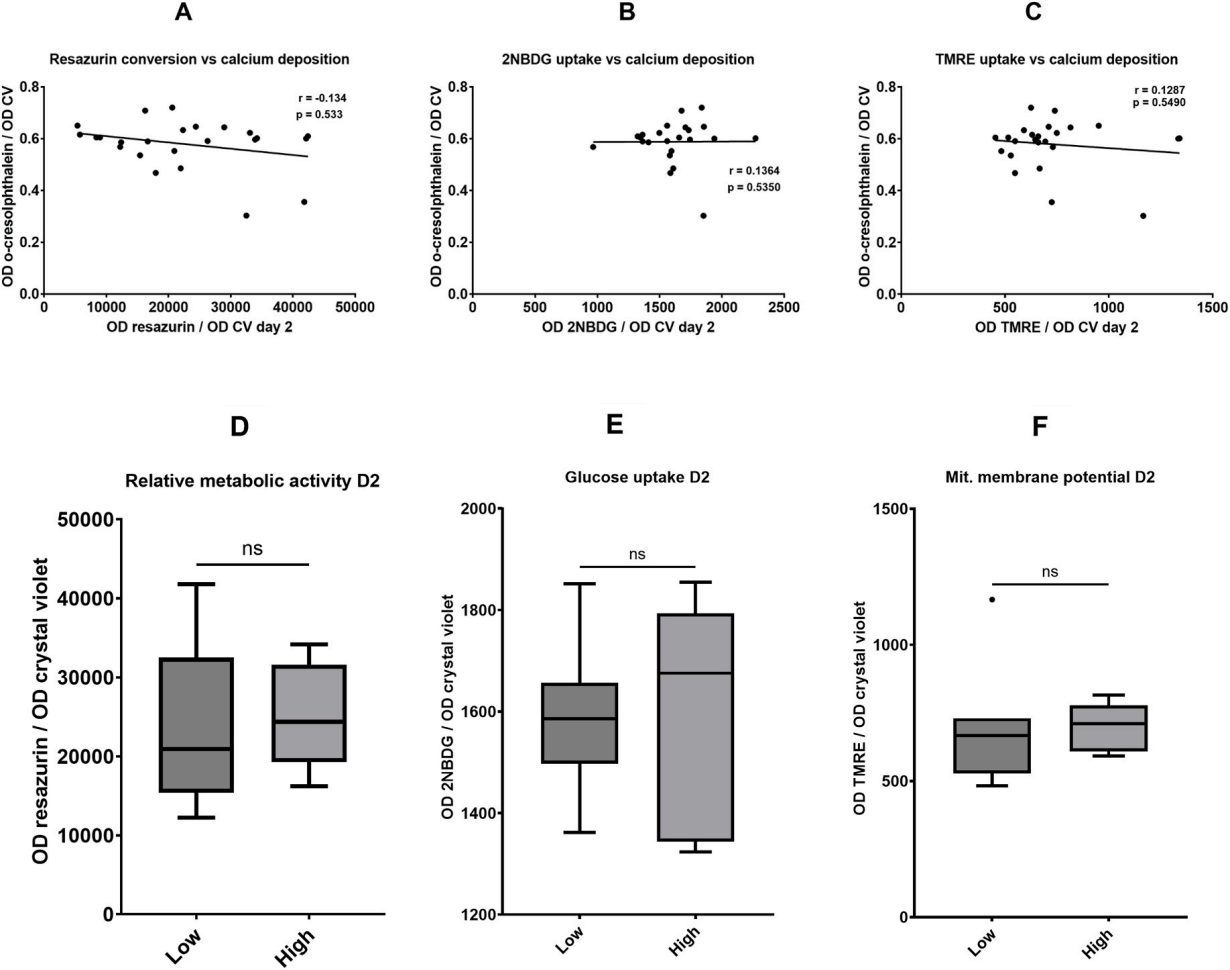
Top panel (A*–*C), Baseline metabolic phenotype does not correlate with donor-dependent mineralization. Spearman correlations (two-tailed) between resazurin conversion (A), glucose uptake (B), or TMRE (C) at day 2 and calcium deposition (cresolphthalein) at day 14. Number of donors n = 24, regression lines shown for visualization only. Bottom panel (D*–*F), metabolic profile of ASCs from low (n = 7) vs high (n = 5) osteogenic potential at baseline (Day 2). (D) relative metabolic activity; (E) glucose uptake; (F) mitochondrial membrane potential (TMRE uptake) assay. Low vs high osteogenic potential groups were defined by day 14 o-cresolphthalein calcium deposition: low = 25th percentile of OD o-cresolphthalein, high = 75th percentile of OD o-cresolphthalein. Statistics: Mann-Whitney *U* test. Data were normalized to cell numbers (optical density of crystal violet) and presented as a box-and-whisker plot. Individual points represent outliers. *p < 0.05, **p < 0.01, ***p < 0.001, ****p < 0.0001.

Using day 14 calcium deposition (o-cresolphthalein) as the primary osteogenic outcome, Spearman correlation analyses revealed no significant association between baseline (day 2) metabolic features and later mineralization. Day 2 summed metabolic activity—quantified via resazurin conversion as a proxy for total cellular reducing capacity and mitochondrial redox state —showed a non-significant negative trend (Spearman *r* = −0.134, *p* = 0.533; Fig. 4A). Similarly, no correlations with day 14 calcium deposition were observed for day 2 glucose uptake (*r* = 0.136, *p* = 0.535; Fig. 4B), which specifically reflects substrate transport, or mitochondrial membrane potential (TMRE) on day 2 (*r* = 0.129, *p* = 0.549; Fig. 4C). This lack of baseline predictive power was further confirmed by stratifying donors into high- and low-osteogenic groups based on their day 14 mineralization levels (75th vs. 25th percentiles). At day 2, no significant differences were observed between these groups regarding their summed metabolic activity, glucose uptake, or mitochondrial membrane potential (Fig. 4D–F).

### 3.4 Late (day 14) metabolic activity correlates inversely with mineralization

**Fig. 5:**
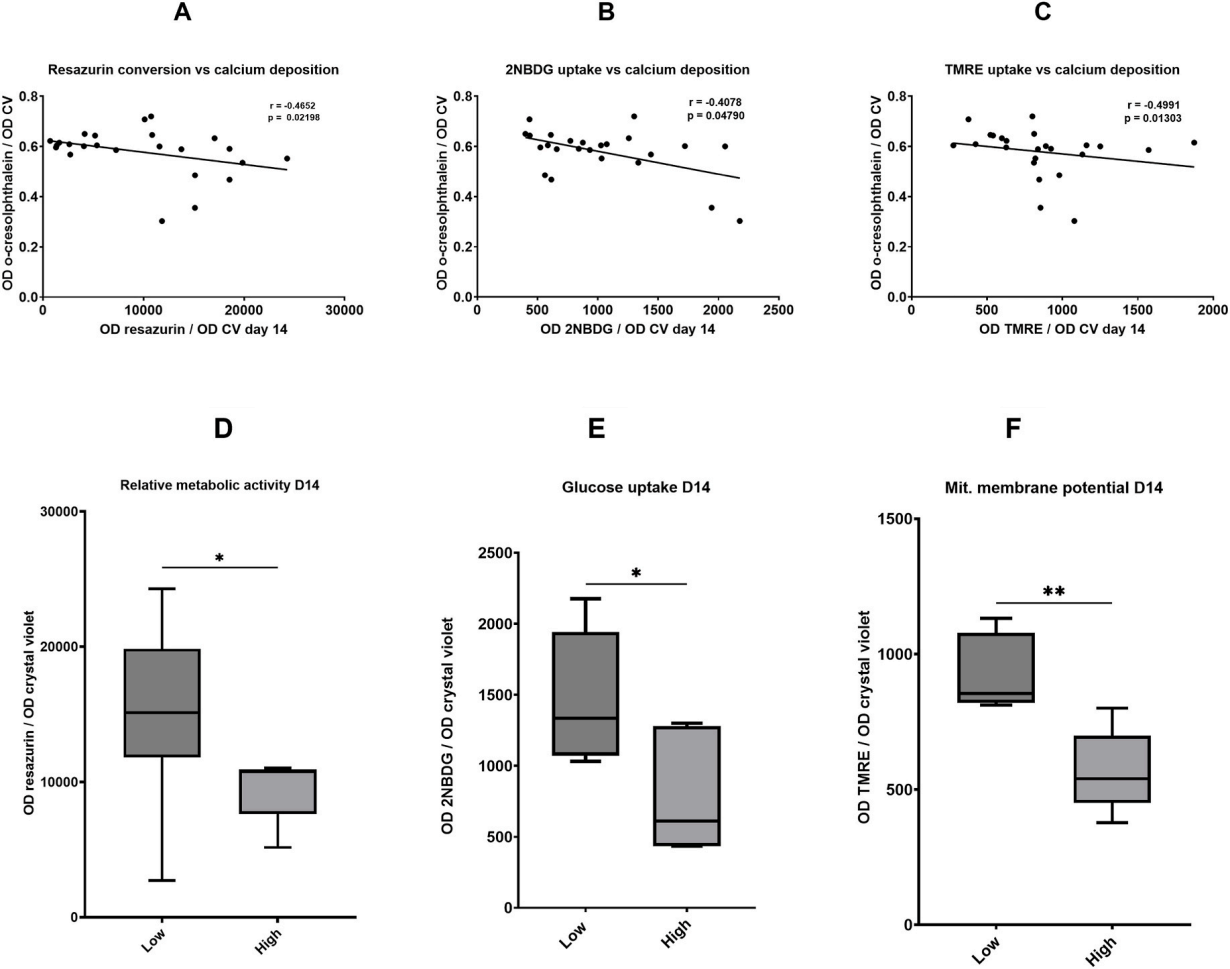
Top panel (A–C), Mineralization is associated with a characteristic late-stage metabolic signature. Spearman correlations (two-tailed) were performed between the primary osteogenic outcome (day 14 calcium deposition via o-cresolphthalein assay) and day 14 metabolic parameters: (A) summed metabolic activity, measured via resazurin conversion as a proxy for total cellular reducing capacity; (B) glucose uptake surrogate (2-NBDG fluorescence); and (C) mitochondrial membrane potential (TMRE uptake). Regression lines are provided for visualization purposes only. Bottom panel (D–F), Metabolic profile of ASC from low (n = 7) vs high (n = 5) osteogenic potential after osteogenic differentiation (Day 14). (D) relative metabolic activity; (E) glucose uptake; (F) mitochondrial membrane potential (TMRE uptake) assay. Low vs high osteogenic potential groups were defined by day 14 o-cresolphthalein calcium deposition: low = 25th percentile of OD o-cresolphthalein, high = 75th percentile of OD o-cresolphthalein. Statistics: Mann-Whitney *U* test. Data were normalized to cell numbers (optical density of crystal violet) and presented as a box-and-whisker plot. Individual points represent outliers. *p < 0.05, **p < 0.01, ***p < 0.001, ****p < 0.0001.

In contrast to baseline, late-stage metabolic measures at day 14 were inversely associated with mineralization. Day 14 metabolic activity by resazurin conversion correlated negatively with day 14 calcium deposition (Spearman *r* = −0.465, *p* = 0.022; Fig. 5A). Similarly, day 14 glucose uptake and day 14 TMRE were also negatively associated with mineralization (2-NBDG: *r* = −0.408, *p* = 0.048; TMRE: *r* = −0.499, *p* = 0.013) (Fig. 5B and C)**.** Exploratory subgroup analysis at day 14 revealed a distinct metabolic divergence. Donors with high osteogenic potential exhibited a significantly lower late-stage metabolic phenotype—characterized by reduced per-cell resazurin conversion and glucose uptake—compared to the low-osteogenic group (Fig. 5D–F). These results indicate that a specialized bioenergetic signature associated with mineralization only emerges during late-stage lineage commitment.

### 3.5 Donor age, BMI, and blood glucose do not show significant associations with mineralization

**Fig. 6:**
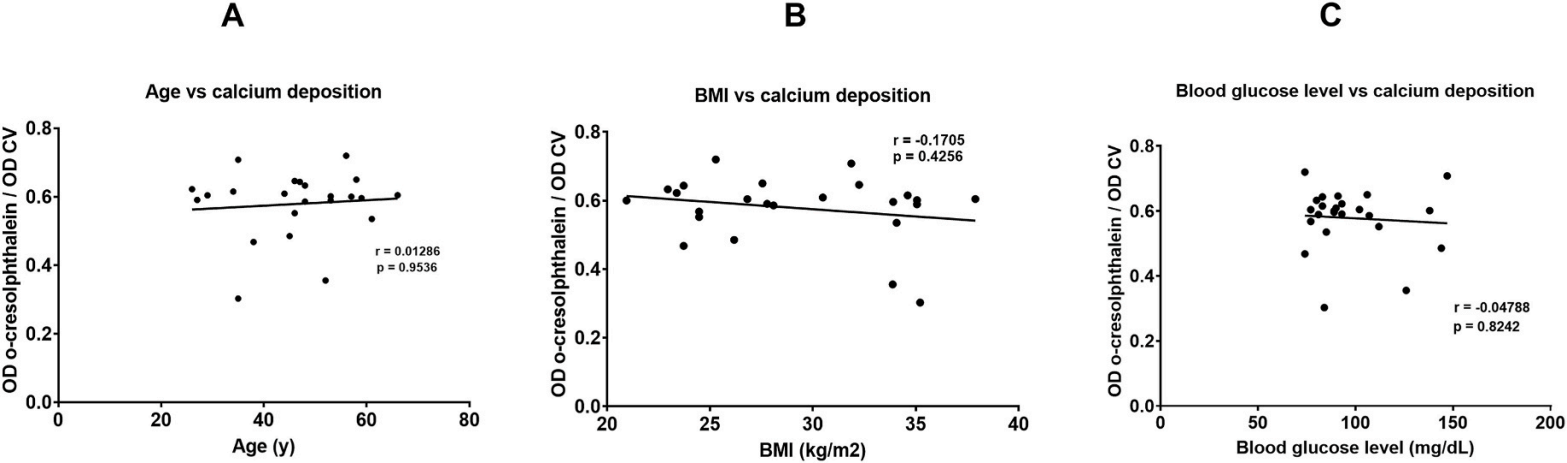
Associations between donor characteristics (age, BMI, and blood glucose level) and ASC mineralization. Spearman correlations between donor age (A), BMI (B), and blood glucose (C) and ASC calcium deposition (day 14). Number of donors n = 23–24, regression lines shown for visualization only.

Clinical patient characteristics available for donors were tested for associations with day 14 calcium deposition. Age did not correlate with mineralization (Spearman *r* = 0.013, *p* = 0.954; Fig. 6A), and BMI (*r* = −0.171, *p* = 0.426; Fig. 6B) and blood glucose (*r* = −0.048, *p* = 0.824; Fig. 6C) showed non-significant negative trends.

## 4 Discussion

This study examined donor dependent variability in ASC osteogenesis by combining osteogenic endpoints with metabolic readouts and basic clinical donor characteristics. As visually and quantitatively demonstrated across the cohort, donors exhibited a broad spectrum of osteogenic and metabolic capacities. Using mineralization (o-cresolphthalein) as the predefined primary outcome, we showed the following: osteogenic induction robustly increased ALP activity, calcium deposition, and secretion of osteogenic proteins across donors; osteogenic conditions were accompanied by higher cell numbers but lower per cell metabolic activity and lower per cell 2-NBDG signal, together with comparatively higher TMRE signal relative to day 14 controls; baseline (day 2) metabolic measures did not predict mineralization at day 14; in contrast, day 14 metabolic parameters showed significant inverse associations with day 14 mineralization; and age, BMI, and blood glucose were not significantly associated with mineralization in this cohort. Overall, these findings suggest that donor dependent osteogenesis is reflected more strongly by a late-stage metabolic phenotype than by early baseline measurements in culture.

The metabolic pattern observed during osteogenic induction — characterized by lower normalized resazurin turnover and reduced glucose uptake (2-NBDG) at day 14 alongside higher TMRE relative to controls — is consistent with the established concept of "metabolic reprogramming" during lineage commitment [11]. This transition represents a shift from the highly glycolytic state characteristic of undifferentiated, proliferative MSCs/ASCs to a more mature phenotype reliant on OXPHOS. While undifferentiated cells prioritize glycolysis to maintain a low-redox environment and minimize oxidative stress, the high energetic demands of bone matrix synthesis and mineral deposition necessitate a profound switch toward mitochondrial respiration and oxidative phosphorylation. Early spectroscopic and metabolic studies have long established that MSCs undergo these distinct metabolic adaptations when placed in osteogenic medium [11,31,32].

The lower per-cell glucose uptake at day 14 does not indicate metabolic dormancy. Instead, it likely reflects a stage-specific shift where cells redirect glucose from the biomass production required for early mitotic expansion toward efficient energy generation and collagen matrix synthesis. In parallel, the higher TMRE signal relative to day 14 controls suggests that ASCs undergoing osteogenesis maintained a high mitochondrial membrane potential to power the ATP-dependent processes of mature osteoblasts, such as protein secretion and ion transport. This high secretory activity was directly evidenced by the elevated release of SPARC and osteocalcin (BGLAP). SPARC is an abundant matrix glycoprotein that binds collagen and calcium to initiate mineral deposition [33], while Osteocalcin (encoded by the *BGLAP* gene) is a mature osteoblast-specific hormone that binds directly to hydroxyapatite during late-phase matrix maturation [34]. Because of these specific biological roles in matrix mineralization, both secreted proteins serve as highly reliable functional markers of osteogenic progression. This remodeling of mitochondrial architecture and substrate preference is not merely a consequence of differentiation but serves as a regulatory driver, where metabolic intermediates influence epigenetic and transcriptional programs required for osteogenic maturation [11,12,14].

Resazurin-based assays mainly reflect cellular reducing capacity and redox state and can decrease during differentiation even when cell number increases, particularly when readouts are normalized to a cell number surrogate such as crystal violet [26]. In this context, the reduced resazurin/crystal violet signal at day 14 was most reasonably interpreted as a change in per cell redox or metabolic state during osteogenic progression rather than reduced overall culture health [26]. The exact effect of osteogenic differentiation on cellular metabolism is defined by a "metabolic switch" where cells transition from a high rate of anaerobic glycolysis to an increased reliance on OXPHOS. This metabolic transition is essential for meeting the high bioenergetic demands of extracellular matrix synthesis, secretion, and mineralization. As cells exit the cell cycle and commit to the osteoblast lineage, they reduce their reliance on the pentose phosphate pathway and glycolytic flux that previously fueled rapid biomass production during early mitotic expansion [11,15].

Glucose handling and general energy metabolism are increasingly viewed as active components of osteoblast lineage biology. Intracellular energy metabolism dynamically interacts with signaling pathways to drive differentiation; for instance, glucose uptake has been linked to RUNX2 accumulation, while AMPK signaling has been implicated as a crucial regulator of the balance between osteogenesis and adipogenesis in both bone marrow and adipose-derived MSCs [13,18,19,35,36]. Our results do not conflict with this framework. Instead, the reduced normalized 2-NBDG signal at day 14 might reflect stage specific remodeling of transporter activity per cell, changes in intracellular processing of the probe, or a shift in substrate preference at later stages of differentiation toward mitochondrial oxidation and matrix production demands [[11], [12], [13], [14]]. Because 2-NBDG and TMRE are proxy readouts, these interpretations should be tested in future work using mechanistic metabolic assays, for example Oxygen Consumption Rate (OCR) or Extracellular Acidification Rate (ECAR), and complementary mitochondrial measures [12,35].

A key finding is the inverse relationship between late-stage metabolic phenotype and mineralization. Donors with stronger calcium deposition showed lower resazurin conversion, and similar negative associations were observed for day 14 2-NBDG uptake and TMRE measures. One plausible biological explanation is that donors whose ASCs mineralize more strongly progressed further toward a mature osteogenic state in which per cell reducing capacity and uptake related signals were reduced, while resources were directed toward extracellular matrix production and mineralization [11,14,15]. This view is in line with reports describing dynamic, stage dependent metabolic remodeling during MSC osteogenesis and osteoblast maturation [11,14,35]. This interpretation is supported by the exploratory subgroup analysis in which high mineralizers exhibited lower per-cell metabolic activity, glucose and TMRE uptake at day 14, alongside stronger osteogenic outcomes (Fig. 5D–F). Similar stage-dependent metabolic remodeling is a hallmark of various mesenchymal stromal/stem cell (MSC) populations, including bone marrow-derived MSCs (BMSCs) and dental pulp stem cells (DPSCs). Importantly, *in vitro* studies have demonstrated that human ASCs possess distinct energy metabolic capacities that dynamically adapt depending on whether they are driven toward osteogenic or adipogenic differentiation [37]. These cells follow a conserved bioenergetic trajectory during lineage commitment, where cellular metabolism is dynamically regulated to transition from a glycolytic state in early progenitors to an oxidative state in mature osteoblasts. For instance, research in BMSCs demonstrates that the early expansion phase is fueled by high glycolytic flux to support rapid proliferation, whereas later maturation phases require a switch to OXPHOS to meet the ATP demands of protein secretion and hydroxyapatite deposition. This transition ensures that the cell's energy allocation shifts from biomass production toward the high-energy requirements of synthesizing a mineralized extracellular matrix [11,14,35].

In contrast, baseline (day 2) metabolic measures did not predict mineralization at day 14. This suggests that donor differences in osteogenic performance may become apparent during lineage progression rather than being detectable by early baseline metabolism in these assays. It is also possible that early predictors exist but were not examined using the current readouts and data sampling techniques. Thus, it may require more sensitive approaches such as oxygen consumption, mitochondrial mass, targeted metabolomics, or early transcriptional programs, as well as earlier time points around the onset of lineage commitment [12,35]. For potency related workflows, this distinction is important. Late differentiation associated metabolic signatures may be useful as practical readouts linked to osteogenic performance, whereas early selection may require different biomarkers or multi parameter models [5,12,35].

MSCs are often described as balancing osteogenesis and adipogenesis, with activation of adipogenic programs occurring at the expense of osteogenesis and vice versa [38]. In our cohort, donors were stratified into groups of high and low osteogenic potential based on their mineralization levels at day 14. At the baseline stage (day 2), no significant differences were observed between these groups regarding their metabolic activity, glucose uptake, or mitochondrial membrane potential (Fig. 4D–F). This indicates that early metabolic states in undifferentiated cells did not reliably predict future osteogenic performance in these assays. However, following 14 days of induction, a distinct divergence emerged: high-osteogenic donors were characterized by a significantly lower late-stage metabolic phenotype — marked by reduced per-cell redox activity and glucose uptake — compared to their low-osteogenic counterparts (Fig. 5D–F). One possible interpretation is that low mineralizing donors may be more prone to an adipogenic leaning state under comparable conditions, consistent with an osteogenic and adipogenic trade-off that could also be reflected in metabolism. Unfortunately, we did not directly test this by performing parallel adipogenic differentiation and marker analyses, because the remaining ASC material was insufficient for additional lineage assays. Since this represents a certain limitation of the present study, future approaches should therefore differentiate osteogenic and adipogenic lineages side by side from the same donors, together with metabolic profiling, to determine whether low mineralizers indeed show higher adipogenic propensity and whether the metabolic signature tracks that lineage bias.

Regarding clinical donor characteristics, we found no significant association between age and mineralization in this cohort. This matches the broader human ASC literature, which however remains inconsistent and is influenced by depot, age grouping, donor health status, induction protocols, and selected endpoints [7]. BMI and blood glucose also did not show significant associations with mineralization or with the metabolic readouts examined here. This may reflect limited variation in these clinical parameters, the use of routine random blood glucose values rather than standardized fasting levels, and the fact that systemic blood glucose does not capture factors likely to influence ASC function, such as insulin resistance, chronic inflammation, medication use, or adipose niche signals [6,14]. Larger cohorts with richer metabolic phenotyping and multivariable models will likely be needed to resolve interacting donor effects and identify clinically accessible predictors.

Furthermore, this work has several limitations. First, donor numbers are modest for correlation analyses, and complete clinical metadata were available for only a subset, which limits power to detect small-to-moderate donor effects and increases susceptibility to outliers. Second, the cohort is predominantly female, which may reduce generalizability and may interact with age-related hormonal status [[39], [40], [41], [42], [43]]. Third, we used per-cell normalized fluorescence/absorbance proxies; while scalable and suitable for high-throughput potency-style testing, these measures are indirect and should ideally be complemented by mechanistic metabolism assays (e.g., OCR/ECAR) and lineage markers (gene expression, matrix composition) [12,35].

Despite these limitations, the overall picture is consistent. Donor-dependent ASC osteogenesis is readily measurable and is accompanied by a recognizable late-stage metabolic phenotype, where stronger mineralization is associated with reduced per cell reducing capacity and altered uptake related signals. From a translational standpoint, these results support including metabolic readouts in ASC characterization alongside established identity criteria and functional osteogenic endpoints, while also motivating future work to identify earlier predictive biomarkers that enable donor or cell selection before long differentiation assays are completed [5,12].

## 5 Conclusions

ASCs from different donors displayed robust but variable osteogenic outcomes after 14 days of induction. Osteogenic differentiation was accompanied by increased proliferation and a distinct metabolic remodeling characterized by reduced per-cell metabolic activity and glucose uptake, with altered mitochondrial membrane potential. Baseline (day 2) metabolic features did not predict later mineralization, whereas late-stage metabolic activity was inversely associated with calcium deposition. Donor age, BMI, and blood glucose were not significantly associated with mineralization in this cohort. These results highlight late-stage metabolic profiling as a pragmatic component of ASC osteogenic potency assessment and motivate future work to identify earlier predictive biomarkers of donor-dependent osteogenic performance.

## Ethics declaration

Written informed consent to take part in the study and to publish the article has been obtained from all participants or their legal representatives. The privacy rights of participants have been observed.

This study included organ or tissue donors. This study includes human biological material and consent was obtained from donors, or their next of kin or legal representatives, for use in this study and for publication of the article. The samples used in this research were not sourced from executed prisoners or prisoners of conscience.

This study was performed in compliance with relevant laws, regulatory frameworks and guidelines where the research took place. This study was conducted in accordance with Declaration of Helsinki. This study was approved by the Regional Ethics Committee of the Faculty of Medicine, RWTH Aachen University. (Approval No. EK163/07)

## Declaration of generative AI use

During the preparation of this work, AS used Google Gemini 3 in order to improve the language and readability of the manuscript. After using this tool, the author reviewed and edited the content as needed and takes full responsibility for the content of the publication.

## Funding

AS was supported by a grant from the Interdisciplinary Centre for Clinical Research within the faculty of Medicine at the RWTH Aachen University (project no. OC1-10).

## CRediT authorship contribution statement

**Abdus Sattar:** Data curation, Formal analysis, Investigation, Methodology, Visualization, Writing – original draft. **Justus P. Beier:** Writing – review & editing. **Tim Ruhl:** Conceptualization, Formal analysis, Investigation, Methodology, Supervision, Writing – review & editing.

## Declaration of competing interest

The authors declare that they have no known competing financial interests or personal relationships that could have appeared to influence the work reported in this paper.

## Data Availability

The datasets generated and/or analyzed during the current study are available from the corresponding author upon reasonable request.

## References

[1] Barba M., Di Taranto G., Lattanzi W. (2017). Adipose-derived stem cell therapies for bone regeneration. Expert Opin. Biol. Ther..

[2] Qin Y. (2023). An update on adipose-derived stem cells for regenerative medicine: where challenge meets opportunity. Adv. Sci..

[3] Dominici M. (2006). Minimal criteria for defining multipotent mesenchymal stromal cells. The international society for cellular therapy position statement. Cytotherapy.

[4] Zhu M. (2013). Manual isolation of adipose-derived stem cells from human lipoaspirates. J. Vis. Exp..

[5] Deskins D.L. (2013). Human mesenchymal stromal cells: identifying assays to predict potency for therapeutic selection. Stem Cells Transl. Med..

[6] Beylerli O. (2024). Donor variability in adipose tissue-derived stem cells: implications for the clinical efficacy of autologous fat grafting. Exploration Med..

[7] Sattar M.A. (2024). Association between donor age and osteogenic potential of human adipose stem cells in bone tissue engineering. Curr. Issues Mol. Biol..

[8] Badimon L., Cubedo J. (2017). Adipose tissue depots and inflammation: effects on plasticity and resident mesenchymal stem cell function. Cardiovasc. Res..

[9] Collon K. (2023). Influence of donor age and comorbidities on transduced human adipose-derived stem cell in vitro osteogenic potential. Gene Ther..

[10] Shyh-Chang N., Daley G.Q., Cantley L.C. (2013). Stem cell metabolism in tissue development and aging. Development.

[11] Shum L.C. (2016). Energy metabolism in mesenchymal stem cells during osteogenic differentiation. Stem Cell. Dev..

[12] Bispo D.S.C. (2025). Metabolic markers detect early ostedifferentiation of mesenchymal stem cells from multiple donors. Stem Cell Res. Ther..

[13] Wei J. (2015). Glucose uptake and Runx2 synergize to orchestrate osteoblast differentiation and bone formation. Cell.

[14] Donat A. (2021). Glucose metabolism in osteoblasts in healthy and pathophysiological conditions. Int. J. Mol. Sci..

[15] Stein G.S., Lian J.B. (1993). Molecular mechanisms mediating proliferation/differentiation interrelationships during progressive development of the osteoblast phenotype. Endocr. Rev..

[16] Tyurin-Kuzmin P.A. (2020). Metabolic regulation of mammalian stem cell differentiation. Biochemistry (Mosc.).

[17] Pattappa G. (2011). The metabolism of human mesenchymal stem cells during proliferation and differentiation. J. Cell. Physiol..

[18] Kanazawa I. (2018). Osteoblast AMP-activated protein kinase regulates glucose metabolism and bone mass in adult mice. Biochem. Biophys. Res. Commun..

[19] Ye J. (2021). The interaction between intracellular energy metabolism and signaling pathways during osteogenesis. Front. Mol. Biosci..

[20] Bourin P. (2013). Stromal cells from the adipose tissue-derived stromal vascular fraction and culture expanded adipose tissue-derived stromal/stem cells: a joint statement of the International Federation for Adipose Therapeutics and Science (IFATS) and the International Society for Cellular Therapy (ISCT). Cytotherapy.

[21] Lingens L.F. (2022). The effect of hypergravity, hyperbaric pressure, and hypoxia on osteogenic differentiation of adipose stem cells. Tissue Cell.

[22] Zhang H.X., Du G.H., Zhang J.T. (2004). Assay of mitochondrial functions by resazurin in vitro. Acta Pharmacol. Sin..

[23] Zou C., Wang Y., Shen Z. (2005). 2-NBDG as a fluorescent indicator for direct glucose uptake measurement. J. Biochem. Biophys. Methods.

[24] Crowley L.C., Christensen M.E., Waterhouse N.J. (2016). Measuring mitochondrial transmembrane potential by TMRE staining. Cold Spring Harb. Protoc..

[25] Ruhl T. (2024). GPR55 inhibits the pro-adipogenic activity of anandamide in human adipose stromal cells. Exp. Cell Res..

[26] Kueng W., Silber E., Eppenberger U. (1989). Quantification of cells cultured on 96-well plates. Anal. Biochem..

[27] Valley M.P. (2016). A bioluminescent assay for measuring glucose uptake. Anal. Biochem..

[28] Lingens L.F. (2022). The effect of hypergravity, hyperbaric pressure, and hypoxia on osteogenic differentiation of adipose stem cells. Tissue Cell.

[29] Bessey O.A., Lowry O.H., Brock M.J. (1946). A method for the rapid determination of alkaline phosphates with five cubic millimeters of serum. J. Biol. Chem..

[30] Gitelman H.J. (1967). An improved automated procedure for the determination of calcium in biological specimens. Anal. Biochem..

[31] Sabini E. (2023). Oxidative phosphorylation in bone cells. Bone Rep.

[32] Reyes J.M. (2006). Metabolic changes in mesenchymal stem cells in osteogenic medium measured by autofluorescence spectroscopy. Stem Cell..

[33] Kaneto C.M., Lima P.S., Zanette D.L. (2016). Osteoblastic differentiation of bone marrow mesenchymal stromal cells in Bruck Syndrome. BMC Med. Genet..

[34] Nakamura A., Dohi Y., Akahane M. (2009). Osteocalcin secretion as an early marker of in vitro osteogenic differentiation of rat mesenchymal stem cells. Tissue Eng. C Methods.

[35] Klontzas M.E. (2024). Machine learning and metabolomics predict mesenchymal stem cell osteogenic differentiation in 2D and 3D cultures. J. Funct. Biomater..

[36] Ning K. (2022). Update on the effects of energy metabolism in bone marrow mesenchymal stem cells differentiation. Mol. Metabol..

[37] Meyer J. (2018). Energy metabolic capacities of human adipose-derived mesenchymal stromal cells in vitro and their adaptations in osteogenic and adipogenic differentiation. Exp. Cell Res..

[38] Infante A., Rodríguez C.I. (2018). Osteogenesis and aging: lessons from mesenchymal stem cells. Stem Cell Res. Ther..

[39] Zhu M. (2009). The effect of age on osteogenic, adipogenic and proliferative potential of female adipose-derived stem cells. J. Tissue Eng. Regen. Med..

[40] Bodle J.C. (2014). Age-related effects on the potency of human adipose-derived stem cells: creation and evaluation of superlots and implications for musculoskeletal tissue engineering applications. Tissue Eng. C Methods.

[41] Horinouchi C.D. (2020). Influence of donor age on the differentiation and division capacity of human adipose-derived stem cells. World J. Stem Cell..

[42] Schipper B.M. (2008). Regional anatomic and age effects on cell function of human adipose-derived stem cells. Ann. Plast. Surg..

[43] Park J.S. (2020). Osteoporotic conditions influence the activity of adipose-derived stem cells. Tissue Eng Regen Med.

